# Editorial and Miscellaneous

**Published:** 1864-12

**Authors:** 


### EDITORIAL AND MISCELLANEOUS.

LANCASTER, O., Nov. 20th, 1864.

*Messrs. Editors:*—Mrs. B., a healthy, laborious woman, mother of four children, menstruated the 4th of July the last time. On the 22d of Sept. following, called my attention to a tumor in the left groin, painless yet dragging. She had slight hemorrhage, and after an examination that diagnosed Ovarian tumor, (not entirely satisfactory,) was not seen until Oct. 3d; then slight hemorrhage and pain in uterus. This continued with variations until the 17th, when finding tampon, astringents and rest unavailing to prevent hemorrhage, I gave ergot, which induced strong uterine contractions and delivered a fœtus, together with four quarts of hydatids.

The child was well-formed, and from maceration appeared as if it had lost vitality two or three weeks. I have had cases of Hydatids, but never before with a fœtus. Is this unusual? I have no means of investigation, and write for information. At the period of the birth the uterus had the size and form of a seven months' conception, not the form exactly, as the weight and size was on the left, and there, no doubt, were the hydatids. Was their presence incompatible with full gestation? is the question of interest. Please answer in the *Journal*.

Yours, TOM O. EDWARDS.

[Uterine hydatids are formed only in the impregnated uterus. They originate from the perverted growth of the villi of the chorion. These villi increase by a process of germination very similar to the roots of a tree. Under normal plastic influences a due proportion is preserved between the increase of the nutrient and depurative powers of the chorion and decidua, and the requirements of the enlarging embryo. But it sometimes happens that this growth of the villi is abnormal, and the cells they contain, increase in size and become dropsical. These constitute uterine hydatids. When recent, these hydatids appear like vesicles filled with a transparent fluid, and are either round, oblong or pyriform in shape. Some are borne upon pedicles, others grow from the walls of larger hydatids. Mettenheimer asserts that on the walls of the primary vesicles, buds appear, and develop separate hydatids, just as the buds protrude from the healthy villi, to produce by normal growth new villosities.

The hydatigenus degeneration of the chorion usually occurs early in gestation. As a consequence nutrition is diverted from the embryo, and it dies. This may explain why a fœtus is not usually present when hydatids are expelled, or as Ramsbotham and Montgomery believe, a portion of the placenta may be left in the uterus after a natural delivery, and form the starting point of a hydatid growth.

In some instances of twin conception one fœtus has disappeared under the influence of hydatigenus degeneration, while the other has continued healthy up to the full term. It is related of the celebrated Beclard that he was born under these circumstances.

If the villi of a portion, only, of the chorion become dropsical, the embryo may still be developed into the fœtus, but a time arrives when the nourishment supplied is deficient, and the fœtus dies.

The hydatids are frequently cast off before the full term of gestation, not always, however, for in some instances they are retained in the uterus long after the term. Two reasons may be given for the early contractions of the uterus and the expulsion of its contents, under these circumstances, 1st. The dead fœtus and abnormal contents of the uterus act as a foreign substance, and provoke contractions. 2d. In normal pregnancy the uterus is not distended by its contents, but increases in capacity by a process of physiological growth. In the hydatigenus degeneration of the chorion, the mass increases in size so rapidly that the uterus becomes mechanically distended, and it contracts and frees itself of its pathological contents.

The development of hydatids is often so rapid that, at the fifth month, the abdomen has attained the size it should present at term. The shape of the uterus is frequently altered from the usual pyriform outline, its growth extending laterally and it becomes irregular in form.

A case occurred in the practice of the writer, during the last summer, in which near twelve pints of hydatid formation with the degenerated placenta were discharged; no trace of a fœtus could be found.—Eds.]

---

*Editors Chicago Medical Journal:—*

In the August number of the *Journal* I notice an article, by Dr. Slayter, "On the Treatment of Healthy Ulcers of the Extremities by Sealing," or by the application of narrow strips of adhesive plaster around them, the whole covered with thin oiled silk. This mode of treatment, introduced, I believe, by Mr. Baynton, of Bristol, England, I have practiced for at least fifteen years, not only with healthy, but also with indolent ulcers, and can add my testimony to that of Dr. Slayter, in regard to its success.

Instead of oiled silk I use a dressing of simple ointment; but the silk would, no doubt, be equally efficacious, and much neater. The first severe case that I recollect treating in this way, was that of a laboring woman, about forty-five years of age, who had been afflicted for several years with an ulcer on the inner and posterior part of the leg, extending from near the knee to the ankle. It occurred after an attack of erysipelas. Various remedies had been resorted to previous to my attendance, with but partial and temporary relief. Under the adhesive strap mode of treatment, in about three months, she was cured and the ulcer healed. I have had a number of cases since, some equally, and one more severe, treated in a similar manner, and with like success. Dr. Slayter observes that this treatment is not generally resorted to in the West; and if these few hastily written remarks will add to the influence of his article in inducing physicians here to try it, my aim will be accomplished. D. B. TRIMBLE.

